# Elevated high-sensitivity troponin T levels at 1-year follow-up are associated with increased long-term mortality after TAVR

**DOI:** 10.1007/s00392-020-01759-x

**Published:** 2020-10-24

**Authors:** Hatim Seoudy, Moritz Lambers, Vincent Winkler, Linnea Dudlik, Sandra Freitag-Wolf, Johanne Frank, Christian Kuhn, Ashraf Yusuf Rangrez, Thomas Puehler, Georg Lutter, Peter Bramlage, Norbert Frey, Derk Frank

**Affiliations:** 1 Department of Internal Medicine III, Cardiology and Angiology, University Hospital Schleswig-Holstein Kiel, Arnold-Heller-Str.3, Haus K3, 24105 Kiel, Germany; 2 DZHK (German Centre for Cardiovascular Research), Partner Site Hamburg/ Kiel/ Lübeck, Kiel, Germany; 3 Department of Cardiology and Angiology, Contilia Heart and Vascular Centre Elisabeth-Krankenhaus, Essen, Germany; 4 Department of Medical Informatics and Statistics, University Hospital Schleswig-Holstein, Campus Kiel, Kiel, Germany; 5 Department of Cardiovascular Surgery, University Hospital Schleswig-Holstein, Kiel, Germany; 6 Institute for Pharmacology and Preventive Medicine, Cloppenburg, Germany

**Keywords:** Troponin T, Transcatheter aortic valve replacement, Aortic stenosis, Survival

## Abstract

**Background:**

Elevated pre-procedural high-sensitivity troponin T (hs-TnT) levels predict adverse outcomes in patients with severe aortic stenosis (AS) undergoing transcatheter aortic valve replacement (TAVR). It is unknown whether elevated troponin levels still provide prognostic information during follow-up after successful TAVR. We evaluated the long-term implications of elevated hs-TnT levels found at 1-year post-TAVR.

**Methods and results:**

The study included 349 patients who underwent TAVR for severe AS from 2010–2019 and for whom 1-year hs-TnT levels were available. Any required percutaneous coronary interventions were performed > 1 week before TAVR. The primary endpoint was survival time starting at 1-year post-TAVR. Optimal hs-TnT cutoff for stratifying risk, identified by ROC analysis, was 39.4 pg/mL. 292 patients had hs-TnT < 39.4 pg/mL (median 18.3 pg/mL) and 57 had hs-TnT ≥ 39.4 pg/mL (median 51.2 pg/mL). The high hs-TnT group had a higher median N-terminal pro-B-type natriuretic peptide (NT-proBNP) level, greater left ventricular (LV) mass, higher prevalence of severe diastolic dysfunction, LV ejection fraction < 35%, severe renal dysfunction, and more men compared with the low hs-TnT group. All-cause mortality during follow-up after TAVR was significantly higher among patients who had hs-TnT ≥ 39.4 pg/mL compared with those who did not (mortality rate at 2 years post-TAVR: 12.3% vs. 4.1%, *p* = 0.010). Multivariate analysis identified 1-year hs-TnT ≥ 39.4 pg/mL (hazard ratio 2.93, 95% CI 1.91–4.49, *p* < 0.001), NT-proBNP level > 300 pg/mL, male sex, an eGFR < 60 mL/min/1.73 m^2^ and chronic obstructive pulmonary disease as independent risk factors for long-term mortality after TAVR.

**Conclusions:**

Elevated hs-TnT concentrations at 1-year after TAVR were associated with a higher long-term mortality.

**Graphic abstract:**

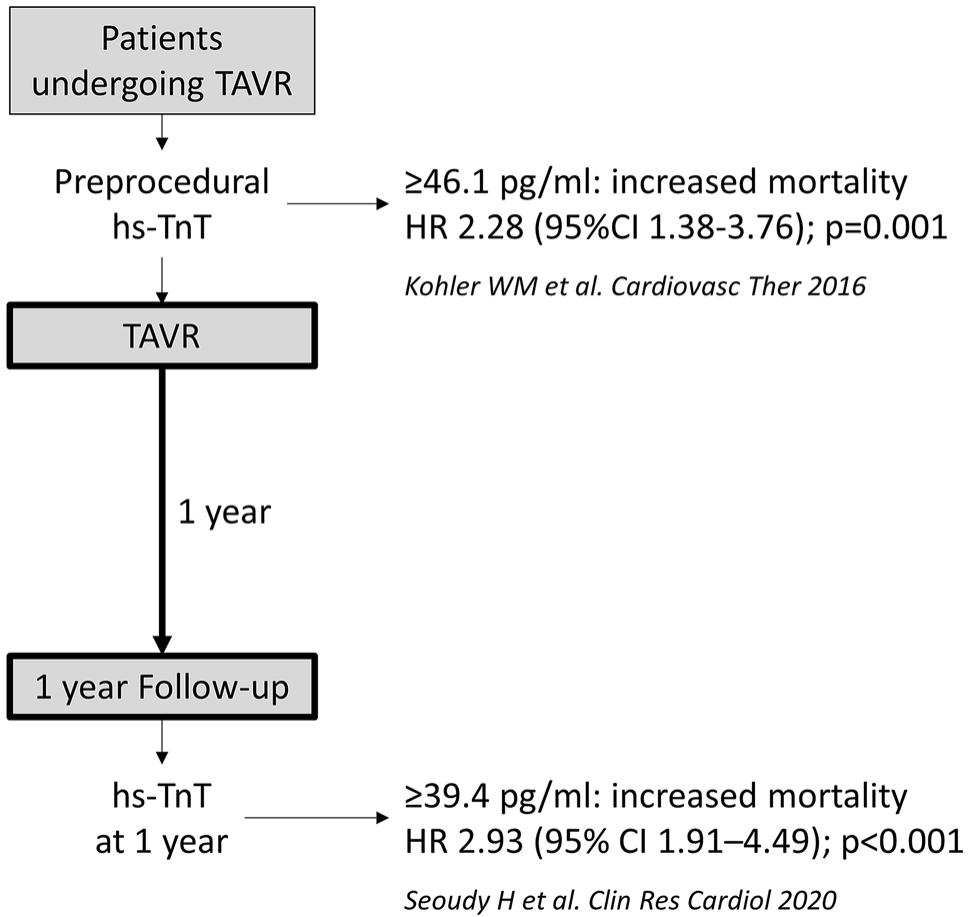

## Introduction

Aortic stenosis (AS) is one of the most common heart valve diseases in the Western world with an estimated prevalence of 3–23%, depending on age [1]. Transcatheter aortic valve replacement (TAVR), which was introduced as an alternative for patients deemed unsuitable for conventional open heart surgery, is now considered the treatment of choice in high-risk patients with severe symptomatic AS, and has also become a viable alternative to surgery in low-to-intermediate-risk patients [2, 3].

In recent years, various biomarkers and multimarker approaches have been studied in the context of AS [4]. High-sensitivity troponin T (hs-TnT) is frequently elevated in patients with severe AS, and we and others have demonstrated that pre-procedural high-sensitive hs-TnT predicts survival after TAVR, with elevated pre-procedural hs-TnT representing an independent predictor of all-cause mortality after TAVR [5–8]. However, the potential role of hs-TnT in risk stratification during follow-up of patients after TAVR remains undefined. Wall stress and subsequent left ventricular (LV) hypertrophy are thought to be major drivers of pre-procedural increased hs-TnT in patients with AS [9], and treatment of severe AS with TAVR leads to an immediate drop in afterload and wall stress in the left ventricle. Thus, elevated hs-TnT values would be expected to decrease shortly after TAVR and definitely by 1-year after treatment.

The objective of our study was to evaluate the prognostic implication of elevated hs-TnT levels found at 1-year after successful TAVR. The hypothesis was that hs-TnT level at 1-year post successful TAVR would still be associated with future survival.

## Methods

### Study design

A total of 953 patients undergoing transfemoral TAVR at our institution for symptomatic severe AS between March 2010 and October 2019 were identified from our TAVR database. Follow-up data including 1-year hs-TnT levels were available for 349 patients who were, therefore, selected for our study. At the follow-up visits, patients were assessed by an experienced cardiologist. Based on the patient’s history, clinical exam and ECG there was no sign of acute myocardial infarction in any of the patients. Patients with an acute myocardial infarction were thus not part of our study.

The primary endpoint of the study was survival time beginning at 1 year after TAVR. The data collection was conducted in accordance with the Declaration of Helsinki and was approved by the local ethics committee at the University of Kiel. All patients provided written informed consent prior to the procedure and data acquisition.

### Data collection

Pre-procedural blood samples and patient data were obtained at baseline and during follow-up. All patients underwent coronary angiography prior to TAVR and percutaneous coronary intervention (PCI) was typically performed more than 1 week before TAVR to minimise the potential influence of revascularisation on hs-TnT levels. Pre-procedural hs-TnT levels were typically assessed 1 day before TAVR, and were measured again after 1 year, during a routine follow-up visit.

Cardiovascular biomarkers (hs-TnT and N-terminal pro-B-type natriuretic peptide [NT-proBNP]) were measured using a system obtained from Roche Diagnostics (“Elecsys^®^ Troponin T high sensitive” [10] and “Roche NT-proBNP” [11]) based on the cobas e801 test module (all Roche Diagnostics, Mannheim, Germany). Patient outcomes were analysed following the Valve Academic Research Consortium-2 (VARC-2) system. Follow-up after discharge usually included an in-person visit at our cardiology outpatient clinic 1–3 months after TAVR, as well as an annual follow-up by either contacting the patients or their general practitioner and cardiologist.

### Statistical analyses

Results are summarised using standard statistical evaluations. Continuous data were expressed as median and interquartile range (IQR), categorical data as counts (percentages). Data were analysed with the Mann–Whitney *U* test and *Χ*^*2*^ test. In case of few observations (frequency less than 10 for an individual cell), Fisher’s exact test was used. Survival data were visualised by Kaplan–Meier curves and compared using log-rank tests.

The cut-off hs-TnT level at 1-year for stratifying risk was determined using receiver operating characteristic (ROC) curve analysis. All pre-procedural variables significantly associated with survival were included in a Cox regression model. Backward selection was based on the likelihood ratio criteria. For each covariate, the proportional hazards assumption was approved by testing for interactions between Schoenfeld residuals and the log‐transformed time using the function “*cox.zph*()” of the “R [*survival*] package”. Results were presented as adjusted hazard ratios (HR) with 95% confidence intervals (CI).

Statistical analyses were performed using the statistical software GraphPad Prism8, and RStudio, version 1.3.1093.

## Results

Among the 349 patients included in the study, 292 had an hs-TnT level < 39.4 pg/mL and 57 had an hs-TnT level of ≥ 39.4 pg/mL at one year.

The median baseline hs-TnT level was 51.2 pg/mL in the high hs-TnT group compared with 18.3 pg/mL in the low hs-TnT group (*p* < 0.001). The high hs-TnT group contained a higher proportion of men compared with the low hs-TnT group (57.9% vs. 41.4%, *p* = 0.028), and had a higher prevalence of a severely decreased estimated glomerular filtration rate (eGFR) < 30 mL/min/1.73 m^2^ (31.6% vs. 4.5%, *p* < 0.001) and peripheral artery disease (26.3% vs. 14.0%, *p* = 0.029). Increased hs-TnT levels were associated with hallmarks of heart failure with preserved ejection fraction (HFpEF) with a higher median left ventricular (LV) mass (253.1 vs. 216.8 g, *p* = 0.005), a higher prevalence of severe diastolic dysfunction (26.3% vs. 10.6%, *p* = 0.004) and a higher median NT-proBNP level (1978 vs. 739.5 pg/mL, *p* < 0.001). The rate of an LV ejection fraction < 35% was higher (12.3% vs. 4.1%, *p* = 0.022) compared with the low hs-TnT group (Table 1).

**Table 1:** Characteristics of patients 1 year after TAVR (baseline for this analysis)

	Total (*n* = 349)	hs-TnT ≥ 39.4 pg/mL (*n* = 57)	hs-TnT < 39.4 pg/mL (*n* = 292)	*p* value
Age [years]	82.0 (78.3–86.1)	82.1 (76.7–86.7)	82.0 (78.4–86.0)	0.904
Female, *n* [%]	195 (55.9)	24 (42.1)	171 (58.6)	0.028
BMI [kg/m^2^]	26.7 (23.9–30.1)	27.2 (23.8–30.5)	26.6 (23.9–30.1)	0.863
CAD, *n* [%]	246 (70.5)	36 (63.2)	210 (71.9)	0.205
COPD, *n* [%]	41 (11.7)	10 (17.5)	31 (10.6)	0.174
CVD, *n* [%]	80 (22.9)	16 (28.1)	64 (21.9)	0.306
Diabetes mellitus, *n* [%]	109 (31.2)	22 (38.6)	87 (29.8)	0.212
Dyslipidemia, *n* [%]	187 (53.6)	26 (45.6)	161 (55.1)	0.195
History of AF, *n* [%]	124 (35.5)	26 (45.6)	98 (33.6)	0.096
Hypertension, *n* [%]	313 (89.7)	50 (87.7)	263 (90.1)	0.634
PAD, *n* [%]	56 (16.0)	15 (26.3)	41 (14.0)	0.029
PAH, *n* [%]	39 (11.2)	9 (15.8)	30 (10.3)	0.250
LVEF				
< 35%, *n* [%]	19 (5.4)	7 (12.3)	12 (4.1)	0.022
35–45%, *n* [%]	38 (10.9)	7 (12.3)	31 (10.6)	0.648
45–55%, *n* [%]	54 (15.5)	10 (17.5)	44 (15.1)	0.689
> 55%, *n* [%]	238 (68.2)	33 (57.9)	205 (70.2)	0.087
RV dysfunction, *n* [%]	60 (17.2)	14 (24.6)	46 (15.8)	0.124
eGFR [mL/min/1.73 m^2^]				
< 30, *n* [%]	31 (8.9)	18 (31.6)	13 (4.5)	< 0.001
30–45, *n* [%]	105 (30.1)	19 (33.3)	86 (29.5)	0.636
45–60, *n* [%]	104 (29.8)	11 (19.3)	93 (31.8)	0.060
> 60, *n* [%]	109 (31.2)	9 (15.8)	100 (34.2)	0.005
Prev. cardiac surgery, *n* [%]	74 (21.2)	11 (19.3)	63 (21.6)	0.860
hs-TnT [pg/mL]	20.8 (13.9–32.3)	51.2 (44.3–69.7)	18.3 (12.7–25.7)	< 0.001
NT-proBNP [pg/mL]	835.5 (378.0–1773.0)	1978 (749.2–4609.0)	739.5 (356.8–1434.0)	< 0.001
AVA [cm^2^]	1.7 (1.4–2.0)	1.7 (1.4–2.0)	1.6 (1.4–1.9)	0.487
MPG [mmHg]	11.0 (8.0–15.0)	9.5 (8.0–14.3)	11.0 (8.0–15.0)	0.445
LV mass [g]	224.2 (184.7–282.0)	253.1 (205.8–301.8)	216.8 (180.9–280.1)	0.005
Severe diastolic dysfunction^1^	46 (13.2)	15 (26.3)	31 (10.6)	0.004
Severe LA enlargement^1,2^	59 (16.9)	14 (24.6)	45 (15.4)	0.092
AR ≥ moderate	3 (0.9)	1 (1.8)	2 (0.7)	0.415
MR III–IV	61 (17.5)	9 (15.8)	52 (17.8)	0.849
TR III–IV	44 (12.6)	5 (8.8)	39 (13.4)	0.512
Transfemoral TAVR	240 (68.8)	37 (64.9)	203 (69.5)	0.533
Balloon-expandable valve	276 (79.1)	48 (84.2)	228 (78.1)	0.298
Mortality rate after 1 year	19 (5.4)	7 (12.3)	12 (4.1)	0.010^3^

Values are presented as counts (percentages) or median (IQR)

*AF* atrial fibrillation, *AR* aortic regurgitation, *AVA* aortic valve area, *BMI* body mass index, *CAD* coronary artery disease, *COPD* chronic obstructive pulmonary disease, *CVD* cerebrovascular disease, *eGFR* estimated glomerular filtration rate, *ES* EuroScore, *hs-TnT* high-sensitivity troponin T, *LV* left ventricular, *LVEF* left ventricular ejection fraction, *MPG* mean pressure gradient, *MR* mitral regurgitation, *NT-proBNP* N-terminal pro-B-type natriuretic peptide, *PAD* peripheral artery disease, *PAH* pulmonary arterial hypertension, *RV* right ventricle, *RVSP* right ventricular systolic pressure, *TAVR* transcatheter aortic valve replacement, *TR* tricuspid regurgitation

^1^Measurements were not available in two patients due to limited acoustic windows or missing data

^2^LA volume index ≥ 40 mL/m^2^ or LA diameter index ≥ 3.0 cm/m^2^

^3^*p* value was calculated using the log-rank test

**Fig. 1:**
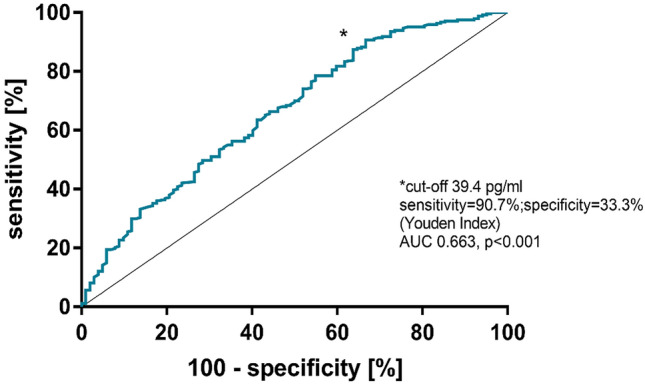
Receiver operating characteristic curve analysis of high-sensitivity troponin T cut-off value. *AUC* area under the curve

**Fig. 2:**
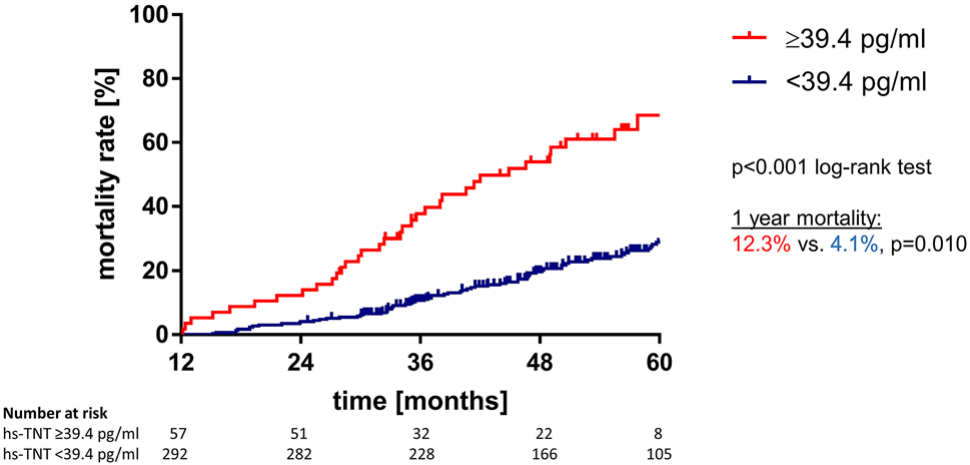
Persistently elevated hs-TnT concentrations at 1 year after TAVR are associated with a higher mortality rates over the long-term. *hs-TnT* high-sensitivity troponin T, *TAVR* transcatheter aortic valve replacement

ROC analysis determined that an hs-TnT level of 39.4 pg/mL was the most appropriate cutoff level for stratifying risk (Fig. 1, Table 2). Of the 349 patients with available 1 year hs-TnT levels, overall mortality rate after 1 year (i.e. within the second year after TAVR) was 5.4% (12.3% vs. 4.1%, *p* = 0.010). The rate of all-cause mortality during the period from 1 to 5 years after TAVR (median follow-up 49.4 months) was significantly higher among those patients who had an elevated hs-TnT level (≥ 39.4 pg/mL) at 1-year post-TAVR compared with those who had a lower 1-year hs-TnT level (*p* = 0.010; Fig. 2).

**Table 2:** Comparison of different cut-offs for hs-TnT [16, 17]

hs-TnT cut-off	Sensitivity [%] (95% CI)	Specificity [%] (95% CI)	Likelihood ratio	Youden-Index
≥ 39.4 pg/mL (*n* = 57)	90.7 (86.4–94.0)	33.3 (24.3–43.4)	1.36	124.0
≥ 52.0 pg/mL (*n* = 27)	96.0 (92.7–98.0)	16.7 (10.0–25.3)	1.15	112.6
> 100 pg/mL (*n* = 6)	99.6 (97.8–99.9)	3.9 (1.1–9.7)	1.03	103.5

*hs-TnT* high-sensitivity troponin T, *CI* confidence interval

Factors that were significantly associated with long-term mortality according to the log-rank test included hs-TnT level ≥ 39.4 pg/mL, NT-proBNP level > 300 pg/mL, eGFR < 60 mL/min, male sex, and chronic obstructive pulmonary disease (COPD) (Table 3). These five factors were also associated with long-term mortality in univariate Cox regression analysis (Table 4).

**Table 3:** Factors assessed at 1-year post-TAVR that were associated with long-term mortality (log-rank test)

	*p* value
hs-TnT ≥ 39.4 pg/mL	< 0.001
hs-TnT ≥ 52.0 pg/mL	< 0.001
Male gender	0.038
COPD	0.022
eGFR < 60 mL/min	0.002
NT-proBNP > 300 pg/mL	0.010

*COPD* chronic obstructive pulmonary disease, *eGFR* estimated glomerular filtration rate, *hs-TnT* high-sensitivity troponin T, *NT-proBNP* N-terminal pro-B-type natriuretic peptide

**Table 4:** Cox regression analysis of factors assessed at 1-year post-TAVR that were associated with long-term mortality

Variable	Univariate analysis		Multivariate analysis	
	HR (95% CI)	*p* value	HR (95% CI)	*p* value
Model 1 (hs-TnT cut-off ≥ 39.4 pg/mL)				
hs-TnT ≥ 39.4 pg/mL	3.60 (2.38–5.45)	< 0.001	2.93 (1.91–4.49)	< 0.001
Male gender	1.50 (1.02–2.22)	0.040	1.64 (1.10–2.44)	0.014
COPD	1.80 (1.08–3.0)	0.024	1.91 (1.14–3.20)	0.015
eGFR < 60 mL/min/1.73 m^2^	2.11 (1.30–3.41)	0.002	1.80 (1.09–2.96)	0.021
NT-proBNP > 300 pg/mL	2.30 (1.20–4.42)	0.012	2.16 (1.12–4.18)	0.022
Model 2 (hs-TnT cut-off ≥ 52.0 pg/mL)				
hs-TnT ≥ 52.0 pg/mL	3.73 (2.20–6.32)	< 0.001	2.82 (1.64–4.84)	< 0.001
Male gender	1.50 (1.02–2.22)	0.031	1.63 (1.10–2.42)	0.015
COPD	1.80 (1.08–3.0)	0.031	1.91 (1.14–3.22)	0.014
eGFR < 60 mL/min/1.73 m^2^	2.11 (1.30–3.41)	0.023	1.95 (1.19–3.19)	0.008
NT-proBNP > 300 pg/mL	2.30 (1.20–4.42)	< 0.001	2.20 (1.14–4.26)	0.019

Results are presented as adjusted hazard ratios (HR) with 95% confidence intervals (CI). Model 1: Global Schoenfeld test: *Χ*^2^ = 3.315, *p* = 0.652. Model 2: Global Schoenfeld test: *Χ*^2^ = 3.320, *p* = 0.669

*COPD* chronic obstructive pulmonary disease, *eGFR* estimated glomerular filtration rate, *hs-TnT* high-sensitivity troponin T, *NT-proBNP* N-terminal pro-B-type natriuretic peptide

Using multivariate analysis, 1-year hs-TnT ≥ 39.4 pg/mL, NT-proBNP level > 300 pg/mL), male sex, eGFR < 60 mL/min/1.73 m^2^ and COPD were confirmed as independent risk factors for long-term mortality after TAVR (Table 4). Notably, an hs-TnT level of ≥ 39.4 pg/mL at 1-year post-TAVR was associated with a 2.9-fold increased risk of long-term mortality (HR 2.93, 95% CI 1.91–4.49, *p* < 0.001) (Model 1). If we replaced the hs-TnT cut-off by ≥ 52.0 pg/mL, the risk increased was largely preserved (HR 2.82, 95% CI 1.64–4.84, *p* < 0.001) (Model 2).

## Discussion

The main finding of our study is that elevated hs-TnT levels at 1-year after successful TAVR are still independently associated with worse long-term survival.

Previous studies have established that elevated pre-procedural hs-TnT levels in patients with AS are prognostic for survival after TAVR [5, 7, 8, 12]. A meta-analysis confirmed that high pre-procedural TnT levels were associated with significant increases in 30-day (*p* = 0.002) and mid-term mortality (*p* < 0.00001) [13]. Studies evaluating the potential prognostic role of hs-TnT measured shortly after TAVR have produced conflicting results [5, 6, 8, 14, 15], although one meta-analysis found that high post-procedural TnT levels were also associated with significant increases in 30-day (*p* < 0.0001) and mid-term (*p* < 0.0003) mortality [13].

With respect to longer follow-up, one study reported that elevation of troponin I (TnI) levels at 3 months post-TAVR was independently associated with an increased risk of 1-year mortality after TAVR (HR 4.4, 95% CI 2.0–9.7, *p* < 0.001) [6]. However, until now, it has not been established whether troponin levels measured later during post-TAVR follow-up can still provide prognostic information about long-term outcomes. To our knowledge, our study is the first to address this question and it has shown that an elevated hs-TnT level at 1-year (defined as ≥ 39.4 pg/mL) after successful TAVR was independently associated with an increased risk of mortality during the subsequent 4 years, with a HR 2.93.

The hs-TnT cut-off level used for risk stratification (≥ 39.4 pg/mL) was calculated using ROC analysis. In our cohort, it reached a sensitivity of > 90% and a sensitivity of > 33%. Choosing a higher hs-TnT cut-off [16, 17] would have increased sensitivity, yet decreased specificity. In addition, using a cut-off of 52 pg/mL or 100 pg/mL would have significantly limited our sample size. Therefore, the cut-off of ≥ 39.4 pg/mL seemed to be reasonable in our cohort. Nevertheless, a cut-off of ≥ 52 pg/mL was also independently associated with further mortality after TAVR. Thus, our analysis indicates that the concept of hs-TnT as a prognostic biomarker after TAVR also holds true when using a population-independent hs-TnT cut-off. The results of the study suggest that monitoring hs-TnT levels during post-TAVR follow-up could help identify those patients at increased risk of mortality after TAVR, which could facilitate individualised management strategies.

Troponin T is a marker of cardiomyocyte damage. Stenosis of the aortic valve causes an increase in LV afterload, leading to LV hypertrophy, which can result in chronic myocardial injury and fibrosis, and thus hallmarks of HFpEF [18]. In fact, increased hs-TnT levels were associated with diastolic dysfunction, LV mass and NT-proBNP. In patients with AS, hs-TnT and hs-TnI levels have been shown to correlate with markers of cardiac remodelling, such as LV mass (reflecting LV hypertrophy), [18–21]. As would be expected, in our study the group of patients with an elevated hs-TnT at baseline had a higher median LV mass than those with a lower hs-TnT level.

NT-proBNP is another marker of myocardial damage. Some studies have found that elevated pre-procedural or early post-procedural levels of NT-proBNP are predictive of mortality in AS patients who undergo TAVR [22–25]. Other studies indicated that NT-proBNP levels that do not decrease after TAVR are predictive of mortality [26, 27]. During longer follow-up, an elevated NT-proBNP level at 30 days post-TAVR, and an increase in NT-proBNP level from baseline to 30 days, have been identified as independent predictors of 1-year mortality [28–30]. However, one of the studies suggested that post-procedural NT-proBNP levels need to be interpreted in the context of the TAVR access route used, with the highest prognostic relevance seen for transfemoral TAVR patients with aortic regurgitation and reduced LV function [25]. Our study found that an NT-proBNP level > 300 pg/mL at 1-year post-TAVR was an independent predictor of long-term mortality (*p* < 0.010) [31, 32].

In addition to 1-year hs-TnT and NT-proBNP levels, we also identified COPD and male sex as independent risk factors for long-term mortality after TAVR. Our findings are consistent with previous studies that have shown that COPD is associated with an increased risk of short- and long-term mortality after TAVR [33–36]. Previous studies on gender differences in outcomes after TAVR have produced conflicting results, but meta-analyses suggest that female gender may be independently associated with better mid-term survival (0.5–1 years) [37–39]. Our findings concerning gender and long-term mortality are consistent with this.

The study has several potential limitations. It was a retrospective, single-centre study with a focus on survival. There may have been a degree of selection bias with respect to the patient population, as only those patients who were healthy enough to do so would have returned for a 1-year follow-up visit. The echocardiography data used in the study were obtained by different physicians and some data were only partially available from the patient files. Our analysis did not specifically address patients who crossed over (i.e., patients who had low hs-TnT values pre-procedure, but high values afterwards, or vice versa), and it may be worthwhile considering such an analysis in the future.

## Conclusion

An increased hs-TnT level at 1-year after TAVR correlated highly significantly with poor long-term survival. This suggests a potential role for hs-TnT in risk stratification during long-term follow-up of patients after TAVR.
